# Artificial Neural Network Prediction of Mechanical Properties in Mycelium-Based Biocomposites

**DOI:** 10.3390/polym17182506

**Published:** 2025-09-17

**Authors:** Štěpán Hýsek, Miroslav Jozífek, Benjamín Petržela, Miroslav Němec

**Affiliations:** 1 Department of Natural Sciences and Sustainable Resources, Institute of Wood Technology and Renewable Materials, BOKU University, Konrad-Lorenz-Straße 24, 3430 Tulln, Austria; 2 Department of Horticulture, Faculty of Agrobiology, Food and Natural Resources, Czech University of Life Sciences Prague, Kamýcká 129, Suchdol, 16500 Prague, Czech Republic; jozifek@af.czu.cz; 3 Faculty of Forestry and Wood Sciences, Czech University of Life Sciences Prague, Kamýcká 129, Suchdol, 16500 Prague, Czech Republic; petrzela@fld.czu.cz (B.P.); nemecmiroslav@fld.czu.cz (M.N.)

**Keywords:** neural network, artificial intelligence, composite material, mycelium, mechanical properties

## Abstract

Mycelium-based biocomposites (MBBs) represent a sustainable alternative to synthetic composites, as they are produced from lignocellulosic substrates bonded by fungal mycelium. Their mechanical performance depends on multiple interacting factors, including the substrate composition, fungal species, and processing conditions, which makes property optimisation challenging. In this study, an artificial neural network (ANN) model was developed to predict two mechanical properties of MBBs, namely internal bonding (IB) and compressive strength (CS). An ANN model was trained on experimental data, using the substrate composition, fungal species, and physical properties of MBBs. The ANN predictions were compared with measured values, and the model accuracy was evaluated. The results showed that the ANN achieved a high predictive accuracy, with coefficients of determination of 0.992 for IB and 0.979 for CS. IB values were predicted more precisely than CS, likely due to microstructural heterogeneities. The heterogeneities were visualised using scanning electron microscopy. Composites produced with *Ganoderma sessile* and *Trametes versicolor* exhibited the highest IB. Interestingly, *Trametes versicolor* achieved the highest CS on virgin wood particles but the lowest values on recycled wood, underlining the strong influence of the substrate quality. The study demonstrates that ANNs can effectively predict the mechanical properties, reducing the number of experimental tests needed for material characterisation.

## 1. Introduction

The development of sustainable materials is driven by the need to reduce the dependence on non-renewable resources. In recent decades, plastics have dominated material production; however, the environmental burden of plastic disposal and the use of synthetic resins in wood-based panels, which often contain volatile organic compounds such as formaldehyde, represent major challenges for recycling and health safety [1]. Mycelium-based biocomposites (MBBs) have emerged as a promising alternative material that addresses these issues by using lignocellulosic (LC) raw materials bonded with fungal fibres without the need for synthetic adhesives [2,3,4].

The binder in MBBs is the fungal mycelium, a three-dimensional network of hyphae with diameters of 2–20 μm, structurally composed of chitin, β-glucans, and glycoproteins [5,6]. During solid-state fermentation, wood-decaying fungi colonise LC substrates such as sawdust, wood particles, straw, and other agricultural residues. The fungus secretes enzymes that decompose cellulose, hemicellulose, and lignin into smaller molecules, which are then metabolised to support hyphal growth [7,8]. This results in a natural fibre network that interconnects substrate particles, forming a porous, lightweight composite with densities typically ranging from 110 to 330 kg/m^3^ [9,10].

A wide spectrum of substrates has been tested, including wood particles, shavings, chips, straw, flax fibres, hemp, cotton residues, coconut fibres, rapeseed straw, coffee waste, and rice husks [1,11,12,13]. The substrate properties, particle size, and nutritional composition strongly influence the fungal growth and the resulting mechanical and physical properties. Larger particles generally improve mechanical strength, whereas fine fractions impair oxygen distribution and hinder colonisation [14,15]. A supplementation with simple sugars and minerals, such as wheat flour, rice bran, gypsum, or calcium carbonate, has been used to enhance fungal growth and improve substrate colonisation [16,17,18]. The choice of the fungal species is another key determinant of MBB performance. Pleurotus species are most frequently employed, followed by Ganoderma, Trametes, and others [17,19,20]. In addition to the final composite properties, criteria such as the growth rate, contamination resistance, and tolerance to environmental conditions are considered in the fungus selection. During production, the surface mycelium often forms a compact fungal skin, which reduces water absorption and enhances durability [17,21,22]. Nevertheless, the bulk MBB typically remains hydrophilic, with water absorption ranging from 40% to 580% depending on the substrate and processing parameters [7,9]. Mechanical properties of MBBs are still modest compared to conventional materials. Reported compressive strengths range from 0.02 MPa for straw-based MBBs to 0.15 MPa for oak sawdust substrates [23].

Although MBBs provide low-density structures with good acoustic absorption, a thermal conductivity of 0.05–0.07 W/m·K, biodegradability, and recyclability, their relatively low strength and high water absorption limit their applications [9,10]. To optimise the performance and tailor MBB properties for specific applications, it is necessary to explore advanced approaches that can predict the relationships between raw materials, fungal strains, physical properties, and mechanical outcomes.

Despite extensive research on the influence of substrates and fungi on MBB performance, the relationships between the composition, physical parameters, and mechanical outcomes remain complex and nonlinear. Conventional statistical methods are limited in capturing these interactions. Therefore, artificial neural networks (ANNs) are promising for predicting MBB properties, as they can learn from data without requiring the explicit modelling of microstructural or chemical details.

Machine learning applications in the field of MBBs are emerging but remain limited. For example, a recent study explored combining experiments and machine learning to optimise MBBs [24]. Another study also discussed the possibility of integrating machine learning in sustainable composite design to predict the properties of MBBs using the substrate type, processing conditions, and additive treatments [25]. However, none of these works to date have developed models that predict internal bonding and compressive strength jointly from a broad set of physical attributes in addition to the substrate and fungal species, nor have they provided detailed feature importance diagnostics.

Therefore, the goal of this study was to develop and validate an artificial neural network model for predicting the mechanical properties of MBBs. Specifically, the model was trained on experimental data using the substrate composition, fungal species, and four measured physical properties of MBBs (water uptake, thermal conductivity, volumetric heat capacity, and thermal diffusivity) as input variables in order to predict the internal bonding and compressive strength. The innovation of this work lies in applying ANN modelling directly to measurable physical properties and categorical inputs, without the need to construct complex models of the microstructure or chemical composition. By doing so, this study not only demonstrates high predictive accuracy but also provides a practical framework that reduces the experimental effort.

## 2. Materials and Methods

Three wood-decaying fungi were used, namely *Ganoderma sessile* (strain: Boston edison, Terrestrial Fungi, USA), *Trametes versicolor* (strain: M 9911, Mycelia, Belgium), and *Ganoderma lingzhi* (strain: M 9724, Mycelia, Belgium). The cultures were maintained on MEA (malt extract agar) mixed from Agar–Agar, Kobe I (Carl Roth GmbH + Co. KG, Karlsruhe, Germany), and 2% malt extract (Carl Roth GmbH + Co. KG, Karlsruhe, Germany).

Virgin wood particles (REF) were obtained from DDL (Lukavec, Czech Republic), recycled wood particles (RW) were obtained from Kronospan CR s.r.o. (Jihlava, Czech Republic), and pulverised kraft lignin was obtained [26].

In order to prepare spawn, the wheat grain was rinsed with water to remove coarse impurities and hydrated to a moisture content of 47 ± 2%, and gypsum was added at 2.5% (d.w.). The mixture was then poured into 1-litre glass bottles with a cotton filter and sterilised for 3 h at 121 °C in an autoclave MLS-3781L (Sanyo Electric Co., Ltd., Osaka, Japan). After cooling, the bottles were inoculated with the respective fungal strains from a Petri dish inside a flow box, Flow FAST H (Faster s.r.l., Rivolta D’adda, Italy), and then incubated at 24 °C for 10 days.

**Figure 1:**
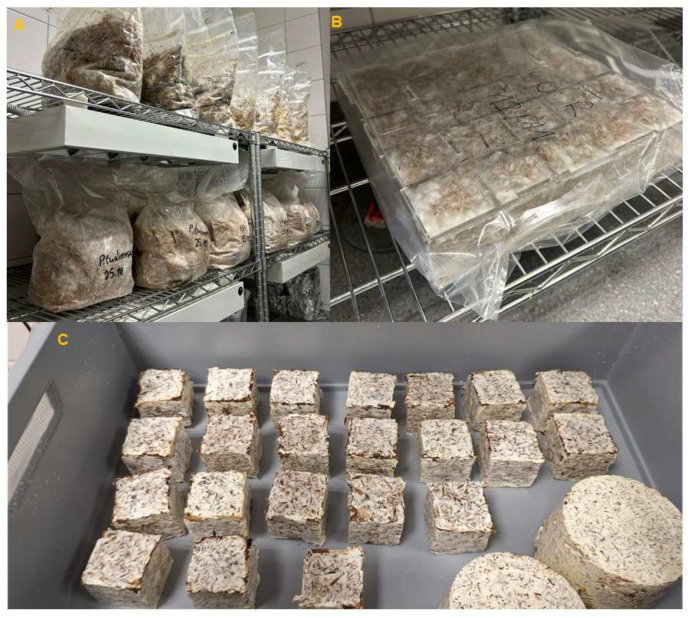
MBB production: colonisation of the substrate (**A**), colonised substrate in cubic forms (**B**), and additional growth phase in EURO crate for growth of fungal skin (**C**).

Three variants of substrate were prepared, namely particles from virgin wood (REF), particles from recycled wood (RW), and particles from recycled wood enriched with lignin (3% dry weight/dry weight) (RWL). The moisture content of substrates was 60%. The substrate was subsequently weighed in 2.5 kg portions into polypropylene (PP) bags (model PP50/SEU4/V40-51, SAC O2 nv, Deinze, Belgium) and sterilised for 3 h at 121 °C in an autoclave MLS-3781L (Sanyo Electric Co., Ltd., Osaka, Japan). After cooling, it was inoculated with grain spawn (10% wet weight/wet weight) of the respective fungal species inside a flow box, Flow FAST H (Faster s.r.l., Rivolta D’adda, Italy), and incubated at 24 °C for 14 days (Figure 1A).

Subsequently, the substrate was manually homogenised and filled into cubic moulds with a side length of 50 mm. For the measurement of thermal insulation, cylindrical samples with a diameter of 100 mm and a height of 70 mm were produced. The filled moulds were incubated for 8 days (Figure 1B). Afterwards, the living composite was removed from the moulds and transferred to a modified EURO crate (lid dimensions 60 × 40 × 18.5 cm) (TBA Plastové obaly s.r.o., Havlíčkův Brod, Czech Republic) with filters for air exchange, where it remained for another 7 days until a strong layer of mycelium was formed on the surface of the composites (Figure 1C). After this period, the composites were dried at 103 °C for 24 h. All growing stages were conducted at a temperature of 24 °C and a relative humidity of at least 95% without light exposure.

The determination of the water uptake was carried out according to the EN ISO 16535 [27] standard. The test specimens were first dried to 0% moisture content in a drying chamber. The drying process lasted 24 h at 105 °C. The dry weight was measured. Subsequently, the samples were immersed in water for 24 h at a temperature of 20 °C and secured with a weight to ensure complete immersion. After this period, the specimens were placed on an inclined plane for 10 min to remove excess water, as specified by the standard, and then the wet mass was measured. The water uptake was calculated based on the difference in mass before and after immersion, using the following equation:

(1) $$Water\;uptake\;\left(\%\right)=\;\frac{m_{wet}-m_{dry}}{m_{dry}}\cdot 100\%,$$

where *m_wet_* is the mass of the test specimen after 24 h of full immersion (kg), and *m_dry_* is the initial mass of the test specimen (kg).

**Figure 2:**
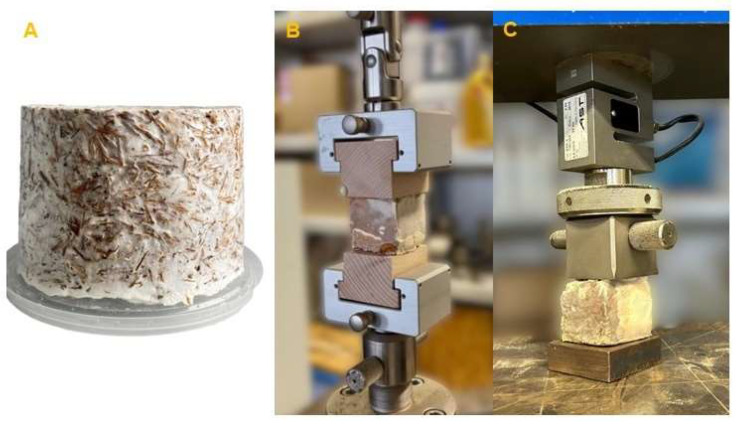
MBB property testing: cylindrical sample for the tests of thermal insulation properties (**A**), internal bonding test (**B**), and compressive strength test (**C**).

Before estimating the thermal insulation properties, the samples were conditioned to a moisture content of 12%. The thermal insulation properties were determined using the ISOMET 2114 measuring device (Applied Precision Ltd., Rača, Slovakia) and the IPS 1105 surface probe. The principle of the measurement is based on monitoring the material’s response to a temperature change over time, caused by a dynamic thermal impulse from the probe. Using this method, the measurement of thermal conductivity (W/(m·K)), volumetric heat capacity (J/(m^3^·K)), and thermal diffusivity (m^2^/s) was conducted. The cylindrical sample for the tests of thermal insulation properties is depicted in Figure 2A.

Internal bonding (tensile strength perpendicular to the plane) was measured according to the adjusted EN 319 standard [28]. The measurements were carried out on the TIRAtest 2850 universal testing machine (TEMPOS, Opava, Czech Republic), and the samples (Figure 2B) were loaded at a constant rate of 10 mm/min until the sample ruptured. Internal bonding was calculated using the following equation:

(2) $$Internal\;bonding\;\left(\mathrm{kPa}\right)=\;\frac{F_{max}}{a\cdot b}\cdot 1000\;\left(\mathrm{kPa}\right),$$

where *F_max_* is the maximal load applied to the sample (N), and *a* and *b* are the initial cross-section dimensions of the sample (mm).

The compressive strength test was conducted according to the EN 826 standard [29], which defines the procedure for determining compressive strength perpendicular to the plane of the board for thermal insulation materials. Samples with dimensions of 50 × 50 × 50 mm were placed between two parallel plates of the UTS 50 testing machine (UTS Testsysteme GmbH, Ulm, Germany) (Figure 2C). Due to surface irregularities, a preload force of 10 N was applied. Once this value was reached, the test specimens were loaded at a constant rate of 5 mm/min. The test ended when a 10% deformation of the specimen was achieved. The compressive strength at 10% deformation was determined using the following equation:

(3) $$Compressive\;strength\;\left(\mathrm{kPa}\right)=\;\frac{F_{10}}{a\cdot b}\cdot 1000\;(\mathrm{kPa}),$$

where *F*_10_ is the force applied to the sample at 10% deformation (N), and *a* and *b* are the initial cross-section dimensions of the sample (mm).

Scanning electron microscopy (SEM) was conducted using a Tescan MIRA3 (TESCAN, Brno, Czech Republic) equipped with an SE detector. The accelerating voltage was set to 12 kV and the working distance to 20 mm. The samples were dried and gold-sputtered.

Two categorical variables (fungal species and substrate type) and four numeric variables, i.e., physical properties of produced MBBs (water uptake, thermal conductivity, volumetric heat capacity, thermal diffusivity), were selected as input variables to predict the mechanical properties of produced MBBs. The predicted mechanical properties were internal bonding and compressive strength at 10% deformation.

The neural network model (NNM) was created using the following parameters: number of neurons per hidden layer was 100, the rectified linear unit function (ReLU; f(x) = max(0, x)) was selected as the activation function for the hidden layer, lbfgs optimizer was used as solver for weight optimisation, the regularisation (L2) was 0.0001, the maximal number of iterations was 200, and the replicable training with fixed random seed was used to ensure reproducibility. The feed-forward multilayer perceptron was trained with the mean squared error objective function:

(4) $$J\left(\theta \right)=\frac{1}{n}\sum_{i=1}{\left(y_{i}-{\hat{y}}_{i}\right)}^{2}+\alpha \left\|W\right\|\begin{array}{c} 2\\ 2 \end{array},$$

where $y_{i}$ is measured values, ${\hat{y}}_{i}$ is predictions, and *W* is the network weights.

Orange Data Mining 3.39.0 software (University of Ljubljana, Ljubljana, Slovenia) was used for neural network modelling (a feed-forward multilayer perceptron neural network was used) as well as for model testing. The two categorical inputs (substrate type and fungal species) were automatically transformed by Orange into one-hot encoded vectors, which ensured that no ordinal relationships were artificially introduced. All numerical variables (water uptake, thermal conductivity, volumetric heat capacity, thermal diffusivity) were standardised using the Preprocess widget to zero mean and unit variance prior to model training. In order to test the neural network model, mean square error (MSE), root mean square error (RMSE), mean absolute error (MAE), mean absolute percentage error (MAPE), and coefficient of determination (R^2^) were calculated. These metrics were calculated both using training data and 10-fold cross-validation (Test and Score widget used).

Due to the modest dataset size and the simplicity of the feed-forward network, model training and prediction were computationally efficient. On a standard laptop, fitting a single model required <1 s, 10-fold cross-validation was completed within a few seconds, and single-sample inference was sub-millisecond. Thus, the proposed ANN model imposes negligible computational cost compared to experimental testing and is suitable for iterative material design workflows.

## 3. Results and Discussion

### 3.1. Physical Properties of MBB

Table 1 presents the average values (including standard deviations) of the input characteristics. It can be seen that all variants of the produced mycelium-based biocomposites reached a lower thermal conductivity than 0.1 W·m^−1^·K^−1^ and therefore are considered to be heat insulation materials. Although an additional growing phase was applied during the composite production in order to produce the fungal skin on the surface of the composites, the water uptake of the produced MBBs is high, ranging from 77% to 168%. Such values of the water uptake of heat insulation materials are, however, comparable with other materials from lignocellulosic resources [30,31,32,33].

### 3.2. Model Performance

**Figure 3:**
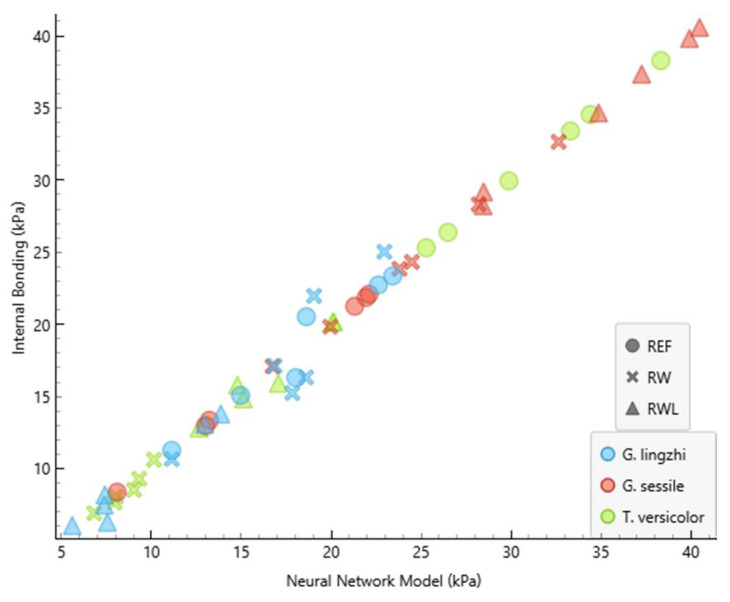
The graphical representation of the measured and NNM values of internal bonding. R2 and MAE are presented in Table 4.

**Figure 4:**
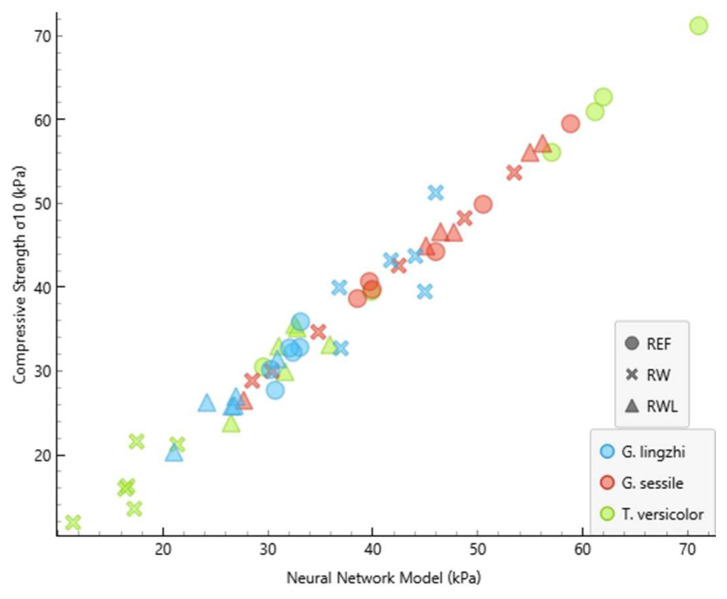
The graphical representation of the measured and NNM values of compressive strength. R2 and MAE are presented in Table 4.

The performance of the developed neural network model (NNM) is summarised in Table 2 and Table 3 and Figure 3 and Figure 4. Table 2 and Table 3 show the comparison of measured and predicted values for the internal bonding and compressive strength, respectively. The predictive accuracy of the NNM is presented in Table 4.

The absolute errors between the measured and predicted values of the internal bonding ranged from 0.011 kPa to 2.927 kPa, while for compressive strength, they ranged from 0.028 kPa to 5.480 kPa. The coefficient of determination (R^2^) reached 0.992 for the internal bonding and 0.979 for the compressive strength. Such high values demonstrate that the developed model can predict the proportion of the variance in the dependent variable from the independent variable to a high extent, confirming the suitability of neural network modelling for predicting MBB mechanical properties. These results indicate that the NNM was able to predict the internal bonding with a greater precision compared to the compressive strength. This outcome is consistent with the fact that internal bonding depends strongly on the strength of the fungal adhesion to the substrate, which is more directly related to the input variables used for training, while compressive strength may be more sensitive to microstructural irregularities not fully captured in the input dataset.

The prediction of both internal bonding and compressive strength exhibited a high level of prediction accuracy (Table 4). This indicates that ANNs can complement experimental approaches by reducing the number of physical tests required for material characterisation, as also highlighted in similar studies on bio-based composites, where ANN models successfully predicted strength-related properties [33]. The extended evaluation metrics of the neural network model are also summarised in Table 4. While the model achieved an excellent performance on the training data, the cross-validation results showed a reduced accuracy, reflected by higher error values and a negative R^2^. This outcome indicates that the relatively small dataset size and structural variability of the composites affect the robustness of the model. Nevertheless, even under cross-validation, the model preserved consistent trends across fungal species and substrates, supporting its potential as a predictive tool when applied to larger datasets.

In terms of experimental results, the highest internal bonding of the produced MBBs was achieved by *Ganoderma sessile*, followed by *Trametes versicolor*. This finding corresponds well with the literature, where both fungal species have been reported as highly effective in forming strong mycelial networks interconnecting lignocellulosic elements, leading to an enhanced mechanical performance [17,19,20]. The fact that the model also reflected these fungal effects in its predictions strengthens the argument that ANN approaches are capable of generalising biological and material interactions into reliable property estimations. The compressive strength values at the 10% deformation obtained in this study were higher or comparable to other thermal insulation materials produced from alternative raw resources, such as bio-based fibreboards and plant-based insulation panels [32,34], confirming the outstanding mechanical performance of the produced MBBs.

To further address interpretability, the relative importance of the input variables was assessed using the RReliefF algorithm (Table 5). The analysis showed that categorical factors, the fungus (0.605 for internal bonding; 0.601 for compressive strength) and substrate (0.570 for internal bonding; 0.563 for compressive strength), were the strongest predictors of both internal bonding and compressive strength. Among the physical properties, the water uptake (0.234 for internal bonding; 0.226 for compressive strength) and thermal diffusivity (0.216 and 0.217, respectively) ranked highest, while thermal conductivity contributed the least.

The graphical representation of the measured and predicted values of the internal bonding and compressive strength is presented in Figure 3 and Figure 4. The clear clustering of predicted and experimental values demonstrates the robustness of the developed neural network model, as also indicated by the high determination coefficients.

Interestingly, the composites produced using *Trametes versicolor* exhibited the highest compressive strength when grown on virgin wood particles (53.5 kPa), whereas the same species cultivated on recycled wood particles resulted in the lowest compressive strength (16.8 kPa) among all variants. This highlights the strong influence of the substrate composition on the performance of MBBs, which is in agreement with previous reports that the chemical composition, contamination level, and particle geometry of lignocellulosic substrates directly affect fungal colonisation and the properties of the resulting composites [8,10,14,35]. Recycled wood may contain adhesive residues and other contamination sources, which can limit the fungal growth of selected species [22,26].

The predictive performance of the ANN model was generally consistent across fungal species and substrates; however, larger deviations were observed in selected cases. For instance, predicted values of the internal bonding and compressive strength for *Ganoderma lingzhi* MBBs grown on recycled wood and the predicted compressive strength of *Trametes versicolor* MBBs grown on recycled wood exhibited higher absolute errors compared to other variants. These deviations can be attributed to structural heterogeneities in the composites that were not reflected in the input variables used for training. Similar challenges in the characterisation of composites have been reported in the literature, where microstructural irregularities, such as the pore distribution, mycelial density gradients, or uncolonized regions, strongly influenced mechanical outcomes but were not captured on a macroscopic level [1,17,19,20]. From a modelling perspective, the observed deviations suggest that including additional microstructural descriptors in the ANN input, such as the porosity, pore distribution, or mycelial surface coverage, could further improve the prediction accuracy.

Overall, the results demonstrate that the application of neural network modelling in MBB research offers the following benefits: (i) supporting the identification of optimal fungal strains and substrate compositions for an enhanced mechanical performance and (ii) providing a predictive tool for material design, thereby reducing the reliance on labour-intensive experimental testing.

### 3.3. Residual Analysis

**Figure 5:**
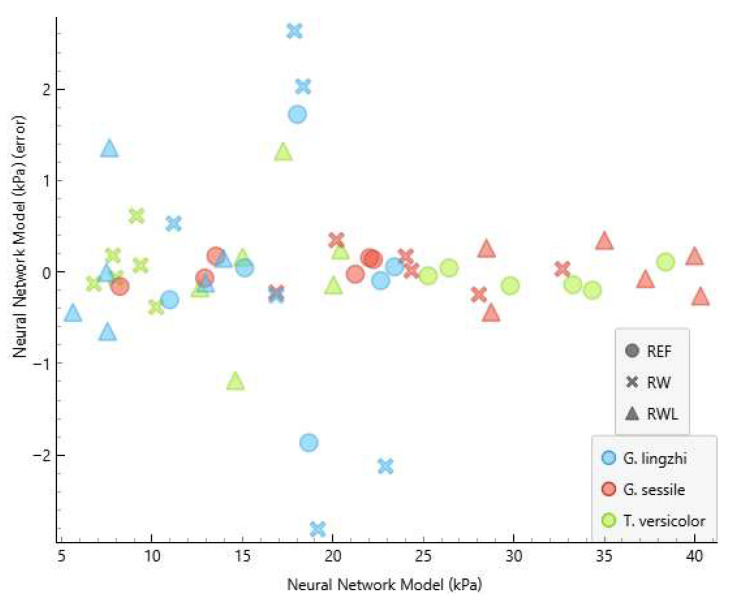
The plot of NNM errors against predicted values for internal bonding.

**Figure 6:**
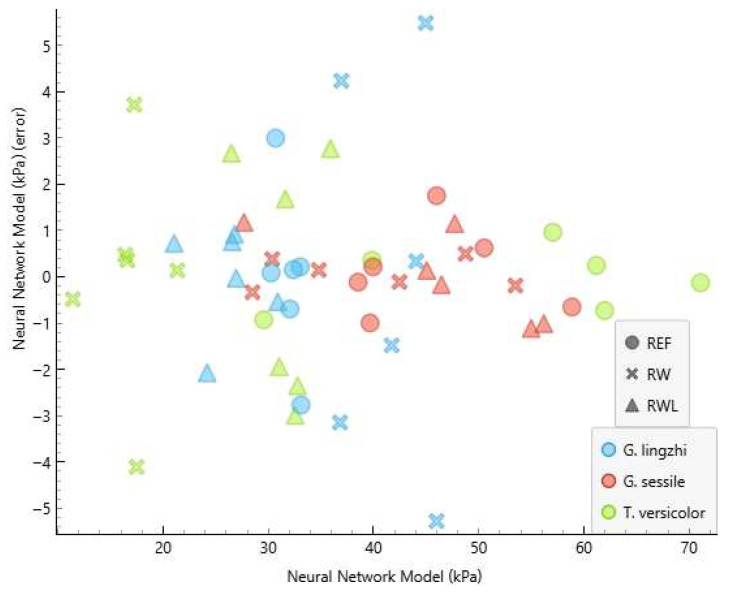
The plot of NNM errors against predicted values for compressive strength.

The predictive errors of the model are graphically depicted in Figure 5 and Figure 6, which plot residuals against predicted values for internal bonding and compressive strength, respectively. For most samples, the residuals are randomly distributed around zero, confirming the absence of a systematic bias in the predictions. However, larger deviations are evident for selected recycled wood composites, particularly *Ganoderma lingzhi* and *Trametes versicolor* variants. This observation supports earlier conclusions that microstructural heterogeneities are not fully captured by the input features but strongly influence compressive strength outcomes.

Compared to conventional statistical or empirical models, which often assume linear independence between input variables, the ANN model demonstrated the ability to capture nonlinear dependencies among the fungal species, substrate type, and physical properties. The advantage of this approach is its predictive accuracy and flexibility, whereas the main limitation is its dependence on sufficient training data. In practice, ANN modelling reduces the number of destructive tests required and can guide the experimental design by highlighting the most influential input features. Thus, ANN methods complement, rather than replace, traditional characterisation.

### 3.4. Microstructural Analysis

**Figure 7:**
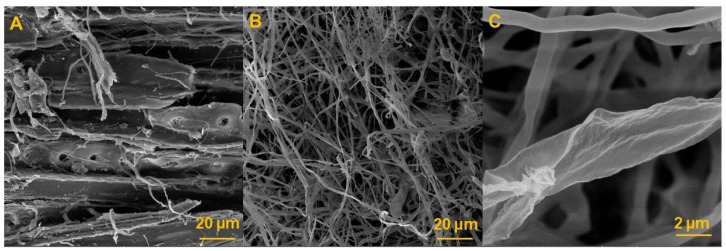
SEM images of the produced MBBs: wood colonised with mycelium (**A**), mycelium composed of individual hyphae (**B**), and detail of one hypha (**C**).

The microstructure of the developed mycelium-based biocomposites was examined by scanning electron microscopy (SEM), and representative images are shown in Figure 7A–C. The analysis confirmed the heterogeneous character of the composites. In Figure 7A, the heterogeneous distribution of wood particles, interconnecting fungal fibres, and pores is visible. Figure 7B depicts the surface of the MBB, where mycelial colonisation results in the coverage of the lignocellulosic substrate. At a higher magnification (Figure 7C), the roughness of an individual fungal hypha can be observed.

It can be concluded that SEM images illustrate several microstructural features that help explain prediction errors observed in the ANN model. In particular, a heterogeneous pore distribution and the incomplete colonisation of some particles may lead to reduced local strength, contributing to higher deviations in compressive strength predictions for recycled wood composites. At a higher magnification (Figure 7C), the hyphal roughness and variability in the surface coverage also indicate potential inconsistencies in the stress transfer across the composite. These features are not captured by the current set of input variables but align with the higher ANN residuals seen in Figure 5 and Figure 6. Incorporating quantitative image-derived descriptors, such as porosity, mycelial density, or fungal skin coverage, into future models could reduce these biases and further stabilise predictions.

### 3.5. Limitations of This Study

Despite the high predictive accuracy of the developed neural network model, several limitations need to be acknowledged. First, the dataset used for the model training and validation was limited in size (six replicates per variant). Although it covered different fungal species and substrates, the number of variants cannot fully represent the broad range of lignocellulosic resources and fungal strains available for MBB production. As shown in previous studies, the variability in the fungal growth and substrate composition can result in significant fluctuations in mechanical properties [8,22,35]. Expanding the dataset with more diverse experimental inputs would likely increase the model robustness and generalizability.

Second, the input variables used in the present model, the substrate composition, fungal species, and selected physical properties, do not capture microstructural characteristics of MBBs. Properties such as the porosity or surface coverage of the fungal skin have been shown to strongly influence mechanical properties and water. Their absence in the input dataset may explain the higher prediction errors observed for some recycled wood composites, where structural irregularities could play a role. Mechanical testing itself introduces sources of variability because in porous materials such as MBBs defects can cause a variability in results. This variability may propagate into the model and affect the prediction accuracy.

The network structure and hyperparameters (one hidden layer with 100 neurons, lbfgs solver, L2 regularisation) were selected based on preliminary testing; more extensive hyperparameter tuning could potentially improve the performance further. Moreover, the input variables were restricted to the substrate type, fungal species, and four physical properties, which do not capture the microstructural heterogeneity. Future work should integrate image-derived descriptors, such as the porosity, pore distribution, and hyphal surface coverage obtained from SEM or µCT, to further stabilise compressive strength predictions, where higher deviations were observed.

## 4. Conclusions

We showed that artificial neural networks can be effectively applied to predict the mechanical performance of mycelium-based biocomposites (MBBs). Using the substrate composition, fungal species, and selected physical properties as input variables, the developed model was trained to estimate the internal bonding and compressive strength at 10% deformation. The high coefficients of determination (0.992 and 0.979, respectively) indicate that the majority of the variability in experimental results can be captured by the model. The results show that internal bonding was predicted more precisely than compressive strength, which may be related to structural heterogeneities not reflected in the model inputs. Nevertheless, the model provided reliable estimations across different fungal species and substrates. In future, such approaches can support the design of tailored, sustainable composites and contribute to the broader application of MBBs in construction, insulation, and packaging, where the mechanical properties are important.

## Figures and Tables

**Table 1:** Arithmetic means and standard deviations of input physical properties.

Fungus	Substrate	N	Water Uptake (%)	Thermal Conductivity (W·m^−1^·K^−1^)	Volumetric Heat Capacity (MJ·K^−1^·m^−3^)	Thermal Diffusivity (m^2^·s^−1^·10^−6^)
*G. lingzhi*	RW	6	153 (8)	0.0825 (0.0008)	0.2347 (0.0099)	0.3519 (0.0124)
*G. lingzhi*	RWL	6	111 (12)	0.0821 (0.0007)	0.2175 (0.0083)	0.3779 (0.0118)
*G. lingzhi*	REF	6	109 (21)	0.0833 (0.0005)	0.2152 (0.0127)	0.3881 (0.0260)
*G. sessile*	RW	6	128 (27)	0.0836 (0.0009)	0.2407 (0.0203)	0.3497 (0.0343)
*G. sessile*	RWL	6	77 (11)	0.0824 (0.0011)	0.2428 (0.0142)	0.3400 (0.0159)
*G. sessile*	REF	6	123 (10)	0.0849 (0.0031)	0.2598 (0.0201)	0.3276 (0.0131)
*T. versicolor*	RW	6	168 (20)	0.0785 (0.0027)	0.2047 (0.0170)	0.3850 (0.0204)
*T. versicolor*	RWL	6	136 (5)	0.0783 (0.0010)	0.2044 (0.0106)	0.3836 (0.0159)
*T. versicolor*	REF	6	108 (7)	0.0751 (0.0041)	0.1605 (0.0080)	0.4693 (0.0373)

Note: Values in parentheses represent standard deviations; N represents sample size per variant.

**Table 2:** Comparison of measured and NNM values for internal bonding.

Fungus	Substrate	Internal Bonding (kPa)	NNM Internal Bonding (kPa)	NNM Error (kPa)
*G. lingzhi*	RW	25.0	23.0	−2.079
*G. lingzhi*	RW	16.3	18.6	2.275
*G. lingzhi*	RW	10.7	11.1	0.474
*G. lingzhi*	RW	17.1	16.9	−0.268
*G. lingzhi*	RW	15.2	17.8	2.597
*G. lingzhi*	RW	22.0	19.0	−2.927
*G. lingzhi*	RWL	8.2	7.4	−0.764
*G. lingzhi*	RWL	6.3	7.6	1.276
*G. lingzhi*	RWL	13.8	13.9	0.077
*G. lingzhi*	RWL	13.1	13.0	−0.119
*G. lingzhi*	RWL	6.1	5.6	−0.453
*G. lingzhi*	RWL	7.5	7.4	−0.102
*G. lingzhi*	REF	23.4	23.4	0.065
*G. lingzhi*	REF	11.3	11.1	−0.140
*G. lingzhi*	REF	20.5	18.6	−1.910
*G. lingzhi*	REF	22.7	22.6	−0.107
*G. lingzhi*	REF	16.3	18.0	1.719
*G. lingzhi*	REF	15.1	15.0	−0.106
*G. sessile*	RW	24.3	24.5	0.156
*G. sessile*	RW	17.1	16.7	−0.355
*G. sessile*	RW	19.8	19.9	0.102
*G. sessile*	RW	23.9	23.8	−0.055
*G. sessile*	RW	28.3	28.2	−0.119
*G. sessile*	RW	32.7	32.6	−0.011
*G. sessile*	RWL	28.2	28.4	0.221
*G. sessile*	RWL	40.6	40.5	−0.105
*G. sessile*	RWL	37.4	37.3	−0.087
*G. sessile*	RWL	39.8	39.9	0.102
*G. sessile*	RWL	29.2	28.5	−0.712
*G. sessile*	RWL	34.7	34.9	0.204
*G. sessile*	REF	13.0	13.0	0.049
*G. sessile*	REF	22.1	22.1	0.013
*G. sessile*	REF	21.9	21.9	0.075
*G. sessile*	REF	13.4	13.2	−0.128
*G. sessile*	REF	8.4	8.1	−0.276
*G. sessile*	REF	21.3	21.3	0.052
*T. versicolor*	RW	8.5	9.0	0.502
*T. versicolor*	RW	10.6	10.1	−0.474
*T. versicolor*	RW	6.9	6.8	−0.082
*T. versicolor*	RW	8.1	8.0	−0.018
*T. versicolor*	RW	9.3	9.3	0.028
*T. versicolor*	RW	7.7	7.9	0.290
*T. versicolor*	RWL	15.9	17.1	1.133
*T. versicolor*	RWL	15.8	14.8	−1.004
*T. versicolor*	RWL	14.8	15.1	0.283
*T. versicolor*	RWL	20.2	20.1	−0.077
*T. versicolor*	RWL	12.8	12.7	−0.157
*T. versicolor*	RWL	20.2	20.1	−0.040
*T. versicolor*	REF	33.4	33.3	−0.096
*T. versicolor*	REF	29.9	29.9	−0.069
*T. versicolor*	REF	38.3	38.3	0.044
*T. versicolor*	REF	25.3	25.3	−0.023
*T. versicolor*	REF	34.5	34.4	−0.139
*T. versicolor*	REF	26.4	26.5	0.112

**Table 3:** Comparison of measured and NNM values for compressive strength at 10% compression.

Fungus	Substrate	Compressive Strength (kPa)	NNM Compressive Strength (kPa)	NNM Error (kPa)
*G. lingzhi*	RW	51.3	46.0	−5.276
*G. lingzhi*	RW	39.5	45.0	5.480
*G. lingzhi*	RW	43.7	44.1	0.336
*G. lingzhi*	RW	43.2	41.7	−1.480
*G. lingzhi*	RW	40.0	36.8	−3.146
*G. lingzhi*	RW	32.7	36.9	4.231
*G. lingzhi*	RWL	20.3	21.0	0.725
*G. lingzhi*	RWL	26.3	24.2	−2.069
*G. lingzhi*	RWL	31.4	30.9	−0.536
*G. lingzhi*	RWL	25.9	26.8	0.921
*G. lingzhi*	RWL	25.8	26.6	0.760
*G. lingzhi*	RWL	27.0	26.9	−0.028
*G. lingzhi*	REF	32.8	33.0	0.215
*G. lingzhi*	REF	32.2	32.4	0.155
*G. lingzhi*	REF	27.7	30.7	2.996
*G. lingzhi*	REF	32.7	32.1	−0.693
*G. lingzhi*	REF	35.9	33.1	−2.767
*G. lingzhi*	REF	30.2	30.3	0.082
*G. sessile*	RW	42.6	42.5	−0.111
*G. sessile*	RW	48.3	48.8	0.496
*G. sessile*	RW	30.0	30.4	0.382
*G. sessile*	RW	28.8	28.5	−0.334
*G. sessile*	RW	53.7	53.5	−0.191
*G. sessile*	RW	34.7	34.8	0.144
*G. sessile*	RWL	57.2	56.2	−1.008
*G. sessile*	RWL	46.6	47.7	1.153
*G. sessile*	RWL	56.1	55.0	−1.112
*G. sessile*	RWL	26.5	27.7	1.179
*G. sessile*	RWL	46.6	46.5	−0.167
*G. sessile*	RWL	44.9	45.1	0.138
*G. sessile*	REF	44.2	46.0	1.752
*G. sessile*	REF	39.7	39.9	0.211
*G. sessile*	REF	49.9	50.5	0.623
*G. sessile*	REF	38.6	38.5	−0.116
*G. sessile*	REF	40.7	39.7	−0.997
*G. sessile*	REF	59.5	58.9	−0.654
*T. versicolor*	RW	16.3	16.6	0.358
*T. versicolor*	RW	11.9	11.4	−0.481
*T. versicolor*	RW	13.6	17.3	3.714
*T. versicolor*	RW	21.6	17.5	−4.106
*T. versicolor*	RW	21.2	21.4	0.141
*T. versicolor*	RW	15.9	16.4	0.475
*T. versicolor*	RWL	23.8	26.5	2.673
*T. versicolor*	RWL	35.5	32.6	−2.989
*T. versicolor*	RWL	33.1	35.9	2.772
*T. versicolor*	RWL	35.1	32.8	−2.350
*T. versicolor*	RWL	29.9	31.6	1.685
*T. versicolor*	RWL	33.0	31.0	−1.940
*T. versicolor*	REF	39.5	39.9	0.356
*T. versicolor*	REF	71.2	71.1	−0.129
*T. versicolor*	REF	62.7	62.0	−0.728
*T. versicolor*	REF	60.9	61.2	0.244
*T. versicolor*	REF	30.5	29.6	−0.927
*T. versicolor*	REF	56.1	57.1	0.962

**Table 4:** Evaluation of the NNM.

Metrics Based on Training Data	MSE (kPa^2^)	RMSE (kPa)	MAE (kPa)	MAPE (%)	R2 (-)
Internal bonding	0.706	0.840	0.460	3.137	0.992
Compressive strength	3.553	1.885	1.291	4.191	0.979
**Metrics Based on Cross-Validation**	**MSE (kPa^2^)**	**RMSE (kPa)**	**MAE (kPa)**	**MAPE (%)**	**R2 (-)**
Internal bonding	204.397	14.297	9.341	46.304	−1.273
Compressive strength	673.462	25.951	19.781	55.694	−2.892

**Table 5:** Ranking of inputs according to RReliefF.

RReliefF	Fungus	Substrate	Water Uptake	Thermal Diffusivity	Volumetric Heat Capacity	Thermal Conductivity
Internal bonding	0.605	0.570	0.234	0.216	0.198	0.131
Compressive strength	0.601	0.563	0.226	0.217	0.208	0.157

## Data Availability

Raw data can be downloaded at the following link: https://doi.org/10.5281/zenodo.17104804.

## Abbreviations

The following abbreviations are used in this manuscript:

ANN	artificial neural network
CS	compressive strength
IB	internal bonding
LC	lignocellulosic
MAE	mean absolute error
MAPE	mean absolute percentage error
MBB	mycelium-based biocomposite
MSE	mean square error
NNM	neural network model
SEM	scanning electron microscopy
R^2^	coefficient of determination
RMSE	root mean square error
